# Waterborne Polyurethane Treated with Flame Retardant Based on Polydimethylsiloxanes and Boron Phenolic Resin for Improving the Char Residue and Anti-Dripping Performance

**DOI:** 10.3390/molecules29235799

**Published:** 2024-12-08

**Authors:** Yadian Xie, Chao Liu, Yujie Wang, Dongmei Bao, Wei Yan, Guoyong Zhou

**Affiliations:** 1 School of Chemical Engineering, Guizhou Minzu University, Guiyang 550025, Chinadmbao@gzmu.edu.cn (D.B.); 2 School of Materials Science and Engineering, Guiyang University, Guiyang 550005, China; lrasyw@163.com

**Keywords:** thermal properties, cure, hybrid, smart materials

## Abstract

Waterborne polyurethane (WPU) was cured with a flame retardant composed of polydimethylsiloxanes and boron phenolic resin. In comparison to unmodified WPU, the heat resistance of the cured WPU film was significantly improved by approximately 40.0 °C, and the limited oxygen index (LOI) increased from 21.9% to 32.6%. The outcomes reveal that the char residue yield of the cured WPU reached a substantial 8.93 wt.% at 600 °C, which is 60 times that of the unmodified WPU. The flame retardant facilitates the creation of char residue with a high degree of graphitization. Furthermore, the total smoke production (TSP), average effective heat of combustion (AEHC), total heat release (THR), and peak heat release rate (pHRR) of the cured WPU were diminished by 66.29%, 48.89%, 28.01%, and 27.96%, respectively, compared to the unmodified WPU. The CO/CO_2_ emission ratio was elevated by 46.32%, and the total flue gas emission was cut by 66.29%, demonstrating a remarkable smoke suppression effect. The cured WPU attained the UL-94 V0 rating without melt-dripping. These results indicate that the combined flame retardants (2.0 wt.%) can endow WPU with outstanding flame retardant properties.

## 1. Introduction

Waterborne polyurethane (WPU) is non-toxic, odorless, and tasteless [1]. However, it is high flammability, and significant dripping behavior during combustion pose a hidden threat to both personal safety and property, limiting its applications in leather processing, textiles, paper manufacturing, architectural coatings, adhesives, and more [2]. Flame retardants can be employed to enhance the char residue yield and reduce dripping in WPU [3].

Flame retardants can be placed into two categories: halogen-based and halogen-free types. However, a large quantity of toxic and corrosive smoke is produced by the polymeric materials decorated with halogen-based flame-retardant during the inflammation process [4,5]. Therefore, halogen-free flame-retardants have gradually become a research hotspot. In recent years, halogen-free flame-retardants, such as the phosphorus-containing flame-retardants have been considered to be effective chemical additives for improving the flame retardance of polymeric materials, including WPU, epoxy resins, acrylic resins, and so on [6]. However, on one hand, phosphorus-containing flame retardant is inclined to slowly absorb moisture and gradually be hydrolyzed to phosphoric acid in the moist air, which seriously limits their practical applications; on the other hand, it is well known that flame retardant will often affect the transparency and the color of WPU. In addition, the phosphorus-containing flame retardant was also found to decrease the glass transition temperature because of an intrinsic plasticizing effect and generalized plasticizing effect [7,8].

Currently, organoboron and organosilicon compounds, known for their low toxicity and odorless properties, are being utilized to develop a novel halogen-free flame retardant. Under these conditions, organoboron- and organosilicon-based flame retardants may serve as promising candidates for achieving halogen-free and phosphorus-free flame retardants that are low in toxicity that are environmentally friendly and highly efficient. Significant efforts have been dedicated to reducing fire hazards associated with WPU, including issues related to dripping and poor mechanical properties in many cases. To date, achieving a WPU with a high char residue yield and anti-dripping capabilities, particularly modified with commercialized flame retardant combinations, remains critically important and challenging for its potential industrial applications.

It is worth noting that WPU cured with an aromatic ring and a B-O bond exhibits superior thermal stability compared to the neat WPU [9,10]. Generally, the design and chemical synthesis of novel flame retardants can be slow, laborious, and inefficient. From this perspective, commercially available flame retardants can be effectively utilized during the development of flame retardant WPU. These commercial flame retardants serve as valuable resources for accelerating the industrialization process. They can be directly employed to fabricate composite flame retardants for potential industrial applications. In this study, the boron phenolic resin, the ‘Y-type’ dangling and linear structure of polydimethylsiloxane, were designed to fabricate the novel flame retardants. A chemical reaction involving the addition of hydroxyls between boron phenolic resin, polydimethylsiloxanes, and isocyanic acid was introduced into the WPU. The flame retardancy and thermal degradation behaviors of WPU composites were investigated by the LOI, the vertical burning test, a cone calorimeter, thermogravimetric analysis (TG), scanning electron microscopy (SEM), and energy dispersion spectroscopy (EDS), etc. In the combustion process, char residue, the melt viscosity, and the strength of the cured WPU were all markedly enhanced [11].

## 2. Results and Discussion

### 2.1. Fourier-Transform Infrared Spectroscopy (FTIR) Characterization

**Figure 1:**
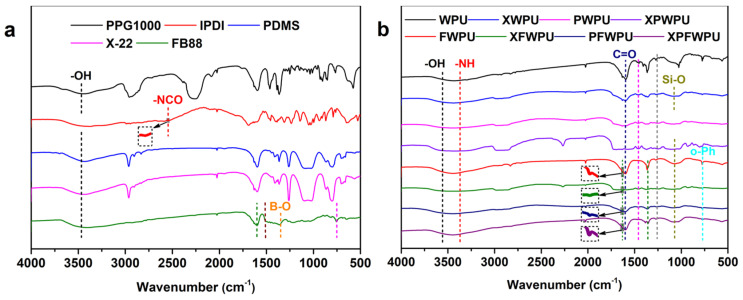
FTIR diagram of the main raw materials (PPG1000, IPDI, PMDS, X-22, and FB88) (**a**), WPU-based polymer (WPU, XWPU, PWPU, XPWPU, FWPU, XFWPU, PFWPU, XPFWPU) (**b**).

To validate the successful preparation of WPU matrix polymers, Fourier-transform infrared spectroscopy (FTIR) was employed to investigate the main raw materials and the WPU based materials. As observed in Figure 1, the strong peak at 3700 cm^−1^~3250 cm^−1^ was attributed to the hydroxyl peak of polypropylene glycol and the hydroxyl absorption peak on the boron phenolic resin backbone, respectively. The strong absorption peaks at 1600 cm^−1^, 1505 cm^−1^, and 750 cm^−1^ were assigned to the characteristic absorption peaks of the benzene ring and its substitution in the boronic phenolic backbone, respectively. In Figure 1b, the absorption peak appearing at 3451 cm^−1^ was confirmed to be the terminal hydroxyl group in WPU. The absorption peaks at 3372 cm^−1^ and 1680 cm^−1^ were attributed to -NH bonds and carbonyl stretching vibration, respectively. The peak at 1380 cm^−1^ was the characteristic absorption peak of the B-O, and 1240 cm^−1^ was due to the stretching vibration peak of C-O-C in WPU. The absorption peak at 1084 cm^−1^ was attributed to the Si-O-Si stretching vibration peak. The characteristic absorption peak of phenyl ring ortho-substitution appeared at 765 cm^−1^ in the boron phenolic resin, and the vibration peak of the benzene ring skeleton appeared at 1600 cm^−1^ and 1400 cm^−1^. The findings suggest that the reaction involving boron phenolic resin, polydimethylsiloxanes and the WPU skeleton has been successfully carried out.

In comparison with the differences between the peak positions and strengths, there may be the following explanations: (1) In general, the absorption of anti-symmetric vibration was greater than that of symmetric vibration. The absorption intensity of telescopic vibration was greater than that of deformation vibration. The boron phenolic resin and polydimethylsiloxanes were the functionalized groups, which can lead to a similar vibration mode of the group and affect the peak strength [12]. (2) The dipole moment of the group was related to the symmetry of the structure. The results reveal that the symmetry of the molecules increases with polydimethylsiloxanes and boron phenolic resin, and decreases with the dipole moment and the absorption peak [13]. (3) The formation of hydrogen bonds will increase the polarization of the bonds and lead to a wider and stronger absorption. The WPU modified with polydimethylsiloxanes was a branched-chain and three-dimensional network structure, and the intramolecular and intermolecular molecules were susceptible to the influence of hydrogen bonds [14].

### 2.2. Emulsion Properties

The appearance of the emulsion, particle size, polydispersity index (PDI), viscosity, and stability indicators are listed in Table S2, respectively. As the content of the flame retardant increased, the average particle sizes of the WPU emulsion rose from 66.33 nm to 147.52 nm. The average particle diameters of the nanoemulsions WPU, XWPU, PWPU, XPWPU, FWPU, XFWPU, PFWPU, and XPFWPU are 66.33 nm, 73.32 nm, 77.16 nm, 87.74 nm, 106.73 nm, 126.70 nm, 110.60 nm, and 147.52 nm, respectively. This increase can be attributed to the interactions between the hydrophilic nature of WPU and the hydrophobic characteristics of the composite flame retardant, which weaken the thickness of the hydrophilic and hydrophobic double layers of the dispersions. Additionally, this increase may also be due to hydrogen bonding between the oxygen and hydroxyl groups of the flame retardant and the ester, urethane, and ureido groups in the polymer, which could enhance the hydration radius of the particles.

The PDI value was maintained between 0.019 and 0.198, indicating that the particle size was evenly distributed without any particle agglomeration or precipitation. The PDI value of the WPU emulsion modified with the composite flame retardant remained at 0.198. This may be attributed to a significant increase in the cross-linking functionality of the boron phenolic resin and the -NCO group, which contributes to hydrophobicity and intermolecular entanglement. Additionally, while the boron phenolic resin enhances the thermodynamic stability of the emulsion, some monomers and prepolymers released during the emulsification and salting processes remain dispersed in the system, resulting in a larger PDI value.

The stability of the emulsion was assessed through high-speed centrifugation. The results indicated that the emulsion was stable. According to the national standard GB/T 6753.3-1986 (methods of test for package stability of paints, Beijing, China, 1986), the WPU emulsion can demonstrate stability, without precipitation and delamination observed six months later.

### 2.3. Water Resistance of the WPU Film

To investigate the hydrophilicity and hydrophobicity of waterborne polyurethane (WPU) films containing various flame retardants, a water contact angle test was conducted. The water contact angle and water absorption rate of the material were evaluated to assess the film’s surface hydrophilicity and hydrophobicity. A larger water contact angle indicates stronger hydrophobicity, while a lower water absorption rate signifies better water resistance. These conclusions can be drawn from a detailed analysis [15].

**Figure 2:**
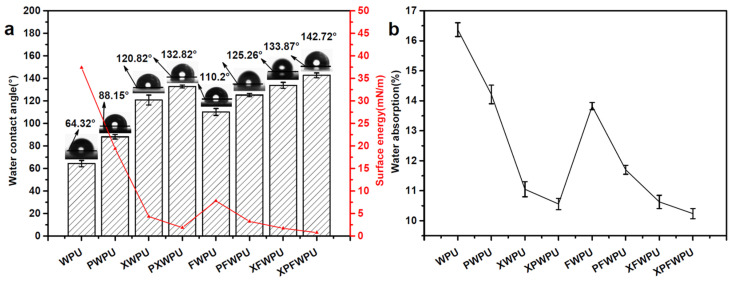
Water contact angle and surface energy of the film (**a**), Water absorption of the film (**b**).

It was shown in Figure 2a that the water contact angle and surface energy of the WPU film decorated with boron phenol and polydimethylsiloxanes (PDMS) have been significantly improved, particularly with composite modification. The water contact angles of the WPU films decorated with PDMS, X-22, and boron phenolic resin were measured at 88.15°, 120.82°, and 110.2°, respectively. In comparison to the neat WPU films, the contact angles of the films decorated with PDMS, X-22, and FB88 increased by 21.83°, 56.5°, and 43.88°, respectively. The corresponding surface energies were 19.39 mN/m, 4.33 mN/m, and 7.8 mN/m, respectively. The hydrophobicity of the cured film was significantly enhanced, with the water contact angles of XPWPU, PFWPU, XFWPU, and XPFWPU being 99.7%, 88.4%, 100.0%, and 114.5% higher than that of the neat WPU. The surface energies for these films were 1.87 mN/m, 3.25 mN/m, 1.72 mN/m, and 0.76 mN/m, respectively.

It is shown in Figure 2b that the water absorption rates of the films modified with PDMS, X-22, and FB88 were 14.21%, 11.05%, and 13.82% higher, respectively, than that of the neat film.

### 2.4. Thermal Stability of the WPU Film

**Figure 3:**
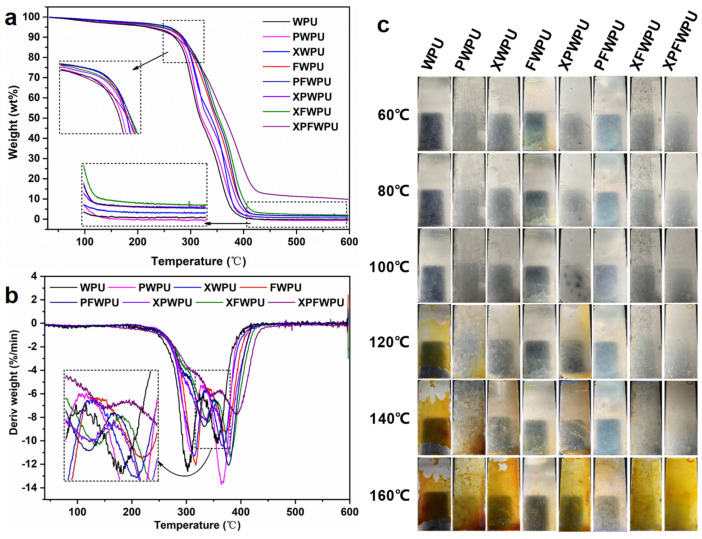
The TG profile of the film: (**a**) DTG curve of the film (**b**), changes in film at different temperatures (**c**).

The thermal stability and char residue-forming ability of polymeric materials are important parameters relating to their flame-retardant performance. The thermal stability of the WPU film was investigated by TG, and the detailed data were summarized in Figure 3 and Table 1. TG analysis was employed to assess the thermal stability and char-forming ability of the WPU [16,17]. The curves of TG and DTG for the WPU were shown in Figure 3a,b, respectively. And the related test data are summarized in Table 1, respectively. There were two decomposition stages in the TG curve of the WPU films; they are 300–350 °C and 350–400 °C, respectively. It can be seen from the TG curve that the temperature of the first decomposition peak (*T*_maxI_) of the WPU decorated with flame retardant was higher than that of neat WPU. In the first decomposition stage, many volatile gasses are released because of the breaking of the bonds between IPDI-derived, hard segments, moieties. The second decomposition stage is attributed to the degradation of the soft segments, with ester-based or ether-based groups.

The *T*_maxI_ of the XPWPU, PFWPU, XFWPU, and XPFWPU were higher 30.6 °C, 33.1 °C, 36.1 °C, and 46.3 °C than that of the neat WPU (302.4 °C), respectively. It was observed that the microphase separation between the soft and hard segments of the WPU was enhanced. As previously mentioned, the thermal decomposition temperature of the soft segment was higher than that of the hard segment [18]. The maximum thermal decomposition temperatures of both the hard and soft segments were shown in Figure 3a. The first decomposition peak, occurring at approximately 280 °C, was attributed to the pyrolysis of polyols, isocyanates, amines, carbon dioxide, and olefins within the hard chain segments of the WPU [19]. Polydimethylsiloxane and boron phenolic resin serve as polyol chemical structures, and these additives were incorporated into the WPU chain segments to extend the carbon skeleton, thereby increasing the carbon content. In comparison to the *T*_maxI_ of WPU modified with a single flame retardant, the *T*_maxI_ of WPU modified with a composite flame retardant showed significant improvement.

The temperature range for the second decomposition stage was approximately 350–400 °C. The maximum temperature of the second decomposition peak (*T*_maxII_) for the WPU modified with PDMS, X-22, and FB88 was higher by 4.1 °C, 10.2 °C, and 10.8 °C, respectively, compared to that of the neat WPU (357.1 °C). The *T*_maxII_ of the WPU films modified with composite materials XPWPU, PFWPU, XFWPU, and XPFWPU was elevated by 16.8 °C, 20.1 °C, 32.9 °C, and 34.2 °C, respectively, compared to the neat WPU. In summary, the thermal decomposition temperature was significantly enhanced.

The WPU modified with polydimethylsiloxanes was influenced by water evaporation during the film formation process. Silicon migrated to the film surface, resulting in an enrichment of silicon–oxygen bonds at the surface. The high bond energy of Si-O contributes to an increase in the thermal decomposition temperature of the film [18]. The decomposition temperature of the film modified with X-22 was 14.9 °C (T10%), 20.5 °C (T50%) higher than that of the film modified with PDMS. The ‘Y-type’ dangling structure of X-22 was more prone to migration to the film surface compared to the straight-chain structure, facilitating the formation of a protective layer. As can be seen from Table 1, the decomposition temperature at each stage was enhanced due to the incorporation of boron phenolic resin. This increase can be attributed to the dehydration curing process of the boron phenolic resin under high-temperature conditions.

This char residue contains a significant amount of air, which is a poor conductor of heat. This property plays a crucial role in heat insulation, preventing heat from being conducted to the surroundings. Above 350 °C, the thermal stability of modified WPU exceeds that of neat WPU. During the combustion process, boron phenolic resin transforms into a polyphenyl network structure, which can bond to aromatic carbon. This transformation produces less flammable gas and enhances heat resistance. The boron-modified WPU improves the thermal stability of WPU due to the high bond energy of boron oxygen bonds. At 600 °C, the char residue of WPU increased from 0.15 wt.% to 8.93 wt.% in a nitrogen atmosphere, attributed to the cured WPU network structure and the incorporation of polyorganosiloxane. This phenomenon may be linked to boron phenolic resin and polydimethylsiloxanes, which can act as precursors, catalysts, or chemical additives that facilitate the formation of char residue and promote its yield during degradation, indicating a synergistic effect between boron phenolic resin and polydimethylsiloxanes. The amount of char residue, or unburned material in WPU, is a critical consideration in the design and preparation of flame-retardant materials. Polydimethylsiloxanes and boron phenolic resin work together to form thermally stable char residue, significantly enhancing the quantity of char residue that blocks heat and mass transfer while inhibiting heat and smoke release in the event of a fire [14]. In the condensed phase, the abundant char residue can prevent the exchange of mass and heat during combustion. This char residue effectively improves the heat resistance and flame retardance of cured WPU. These results demonstrate that this novel combination can significantly enhance the thermal stability and char residue yield of cured WPU.

**Figure 4:**
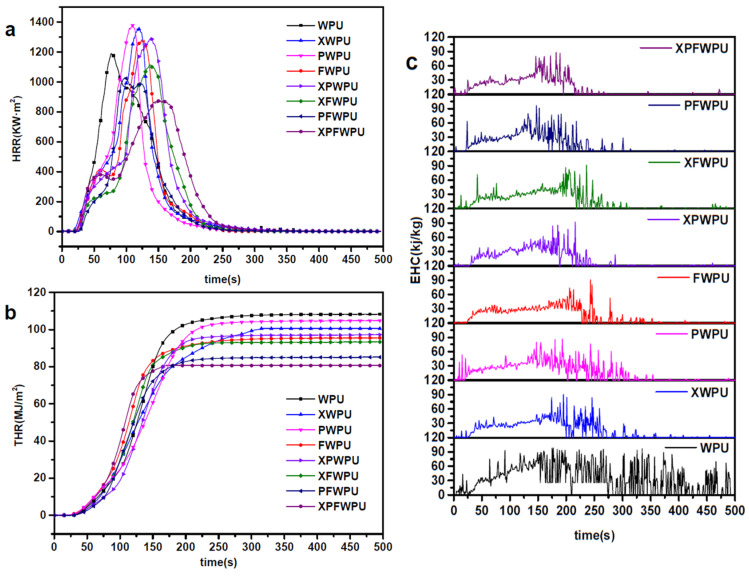
The HRR (**a**), THR (**b**) and EHC (**c**) of the cured WPU films.

Figure 4a shows the effects of the film being subjected to temperatures ranging from 60 °C to 160 °C over a 24 h period (at a heating rate of 10 °C/min). As can be seen from Figure 3c, there was a slight change in the film at 60 °C. However, the WPU-based film began to exhibit a milky white appearance within the temperature range of 80 °C to 100 °C. Being different from the milky white PFWPU, XFWPU, and XPFWPU films, the color of the PWPU, XWPU, FWPU, and XPWPU films gradually transitioned to brown.

At 140 °C, the WPU film transitioned into a viscous state, resulting in a compromised appearance and a noticeable yellowing of the modified films. At 160 °C, the WPU film turned yellow–brown, indicating complete aging. The PWPU, XWPU, and XPWPU films exhibited significant yellowing, deepening of color, and overall aging. In contrast, the FWPU, PFWPU, XFWPU, and XPFWPU films showed increased yellowing, yet their structural integrity remained stable, and their appearance was unblemished. It is evident that the thermal stability of WPU can be progressively enhanced through the simultaneous incorporation of Si-O-Si, phenolic resin, and silicon boron. In summary, the thermal stability of the cured films is significantly higher than that of the neat WPU.

### 2.5. DSC Analysis of the WPU Film

The glass transition temperature (Tg) of the film was shown in Figure S2. The Tg values for PWPU, XWPU, FWPU, PFWPU, XPWPU, XFWPU, and XPFWPU are −36.25 °C, −37.15 °C, −39.91 °C, −37.52 °C, −38.45 °C, −37.88 °C, and −43.77 °C, respectively. These values are lower, by 2.53 °C, 3.43 °C, 6.19 °C, 3.80 °C, 4.73 °C, 4.16 °C, and 10.05 °C, compared to that of the WPU, which has a Tg of −33.72 °C. In comparison to the neat WPU film, boron phenolic resin has been employed to enhance flexibility and rigidity. This modification proved to be more effective than that of the modification with polydimethylsiloxanes. The structure of polydimethylsiloxanes (PDMS) is a linear structure. The flexibility of PDMS molecules helps prevent the embrittlement of the film. The cantilever structure X-22, characterized by its rigidity and ductility, can improve mechanical strength. The Tg of WPU, modified with X-22, is lower than that of the WPU modified with PDMS. The results show that PDMS, X-22, and FB88 can endow the cold tolerance of the film.

### 2.6. Conical Calorimetric Test

Cone calorimeter measurements are increasingly utilized to evaluate the combustion behavior of materials. The cone calorimeter provides essential parameters for assessing the flame retardancy of the materials, including the time to ignition (TTI), the heat release rate (HRR), the peak heat release rate (pHRR), average amounts of CO and CO_2_ generated (ACOY, ACO_2_Y), total smoke production (TSP), the average effective heat of combustion of volatiles (AEHC) and the total heat release (THR). Under a radiated power of 35 kW·m^−2^, the combustion characteristics of the film were studied using a cone calorimeter. The results are shown in Table 2.

Heat release rate (HRR) is one of the important parameters, which is employed to characterize fire intensity and serves as a key basis for evaluating the fire potential of materials. HRR indicates that the more intense the surface heat release of a material, the higher the thermal decomposition rate and the greater the likelihood of fire [20]. As shown in Figure 4 and Table 2, the HRRs of the WPUs modified with the X-22, PDMS, and the novel flame retardant combination were gradually reduced.

The incorporation of the X-22, PDMS, and a novel flame retardant combination increased the time (pHRR) to a higher level, indicating their effectiveness in suppressing fire propagation, presumably by participating in the charring reaction of the WPU matrix. The pHRR values for the XWPU, PWPU, XPWPU, and FWPU films were 1353.55 kW·m^−2^, 1378.46 kW·m^−2^, 1292.22 kW·m^−2^, and 1295.61 kW·m^−2^, respectively, which represent increases of 11.41%, 13.46%, 6.36%, and 6.64% than that of the neat WPU (1214.93 kW·m^−2^). In comparison with the neat WPU pHRRs (1214.93 kW·m^−2^), the pHRRs of the XFWPU, PFWPU, and XPFWPU were significantly reduced. In the same way, the pHRR of the AEHC was also markedly reduced. The AEHC of the WPU, XWPU, PWPU, FWPU, XPWPU, XFWPU, PFWPU, and XPFWPU films was significantly reduced to 1114.30 kW·m^−2^, 1039.04 kW·m^−2^, and 875.24 kW·m^−2^, reflecting decreases of 8.28%, 14.48%, and 27.96%, respectively, compared to the neat WPU.

The results indicate that the THR of WPU modified with the composite flame retardant has significantly improved thermal insulation and flame retardant performance. It is evident from the HRR of WPU modified with X-22 and PDMS individually or in combination that HRR is decreased when compared to modification with boron phenolic resin alone. However, the HRR of modified WPU in the silicon–boron compounding system decreased. The WPU modified with X-22, PDMS, and boron phenolic resin individually does not effectively form a thermal insulation structure to prevent heat transfer. The HRR of the XWPU film is lower than that of the PWPU film, likely due to the Si-O bonds in the ‘Y-type’ structure of X-22, which tend to migrate to the surface and promote the formation of a more effective char residue. The XPWPU, which incorporates silicon–boron composite flame retardance, has better flame retardance than that of the individual modification. The HRR of the WPU modified with the silicon–boron composite system has decreased significantly, indicating that the silicon–boron composite plays a synergistic role in effectively improving the flame retardancy of the WPU.

The THR and total flue gas emissions of the boron phenolic resin-modified WPU film were significantly lower than those of the neat WPU films. This indicates that the addition of boron phenolic resin has a pronounced effect on reducing the HRR of the WPU. On the one hand, phenolic resin has high temperature and flame resistance, and its benzene ring structure can form aromatic carbon compounds and char residue when burned or during high temperature processes, reducing the generation of flammable gasses [21]. On the other hand, due to the presence of boron atoms coming from the boron phenolic resin, it can form glassy carbon with a dense structure during the cracking process, which can effectively prevent oxygen from diffusing, inhibit the further combustion of the film, reduce the HRR and the THR, and improve the flame retardant performance [22]. In a word, the great suppression of the heat, smoke, and toxic effluents confirmed that XPWPU, the silicon–boron composite flame retardant, can remarkably promote fire safety for the WPU matrix.

### 2.7. Smoke Suppression, Flue Gas Release, and Its Toxicity Analysis

The smoke produced during a fire disaster is often more lethal than the fire itself, making it a critical factor in assessing the fire hazards of materials. Additionally, the toxic flue gasses generated from the WPU material during combustion pose a significant threat to both the ecological environment and personal safety [23]. To gain a deeper and more comprehensive understanding of the fire behavior of the cured WPU, we recorded the burning characteristics of the cured WPU, and the neat WPU films were recorded by the cone calorimeter test. This included studying the smoke release rate (SPR), total smoke production (TSP), CO and CO_2_ generation rate (COR, CO_2_R), could also be obtained by the test. At present, the reduction in flue gas emissions and their toxicity represents a crucial direction in flame retardant research [24].

**Figure 5:**
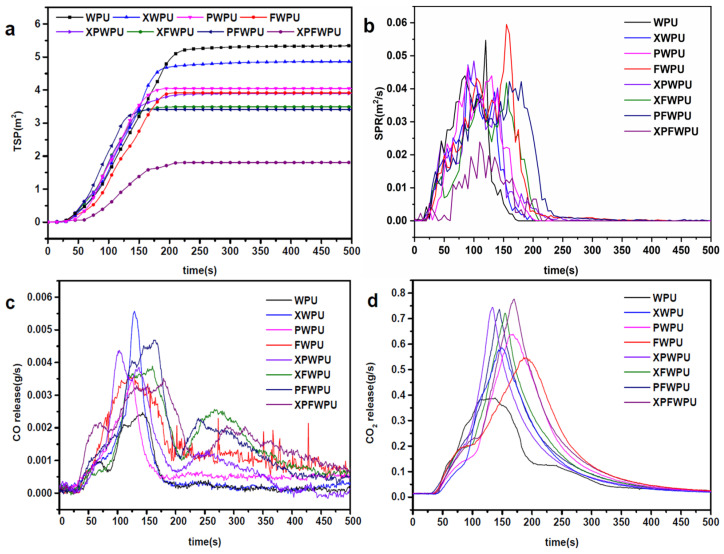
TSP (**a**) and SPR (**b**) of the films and CO generation rate (**c**), and CO_2_ generation rate (**d**).

The TSP value of the cured WPU film was significantly lower than that of the neat WPU film, as illustrated in Table 2 and Figure 5a. Specifically, the TSP values for the cured films, XWPU, PWPU, FWPU, XPWPU, XFWPU, PFWPU, and XPFWPU, were reduced by 9.50%, 24.58%, 27.19%, 27.37%, 34.82%, 36.50%, and 66.29% compared to that of the neat WPU. The average TSP of the cured WPU films was markedly decreased compared to that of the neat WPU film, attributed to the synergistic effects of silicon and boron.

As shown in Figure 5b, before 120 s, the TSP of the cured film was lower compared to that of the neat WPU. After 120 s, as combustion continued, the addition of modifiers resulted in a TSP for the cured film that was higher than that of the neat WPU. The synergistically modified film exhibited a more pronounced effect in inhibiting flue gas release compared to the individually cured films. The TSP of the XWPU film was lower than the total flue gas release of PWPU film, and the TSP of the XPWPU film was lower than that of the films modified with X-22 or PDMS. It can be seen from Figure 5 that the film modified with X-22 was more easily migrated to the surface than that of the PDMS, and the improvement of smoke suppression performance was more obvious. The WPU cured with boron phenolic resin in the cracking process is inclined to form glassy carbon with a dense structure. It effectively suppresses smoke by reducing the emission of toxic flue gases, lowering the overall flue gas output, and enhancing smoke suppression capabilities. The CO and CO_2_ release rates of the cured film were improved, as shown in Table 2 and Figure 5c,d, and the release rates of the CO and CO_2_ in the same period of time were researched. Compared with the whole process, the release rates of CO and CO_2_ are lower during the time 0–200 s. It can be seen that the release amount of the CO_2_ were in principal status in this stage, because this stage involved the formation process of the carbon layer [20]. A total of 200 s later, the release rate of CO_2_ gradually decreased to zero, while the release rate of CO showed an upward trend. With the combustion process, a carbon layer was gradually generated, and the release rate of CO gradually increased. The carbon-forming capacity of the modified film was enhanced to prevent the entry of external combustibles and incomplete combustion intensifies. The CO/CO_2_ values of WPU, XWPU, PWPU, XPWPU, XFWPU, PFWPU, PFWPU, and XPFWPU were 0.0190, 0.0229, 0.0192, 0.0235, 0.0230, 0.0237, 0.0253, and 0.0278, respectively. It can be seen that the flame retardance and smoke suppression effect of the film cured with boron phenolic resin was provided with excellent performance. The nature of the migration of Si-O in the X-22 structure was also recorded; the flame retardance and smoke suppression effect after modification was better than the that of PDMS, the effect of silicon–boron composite modification was outstanding, and the flame retardant and smoke suppression performance of XPFWPU film was similarly outstanding. In summary, boron phenolal, X-22, and PDMS can endow the film with different degrees of heat resistance and smoke suppression performance, and the effect of the silicon–boron collaborative modification function was overall outstanding.

### 2.8. Surface Morphology and the Analysis of Char Residues

**Figure 6:**
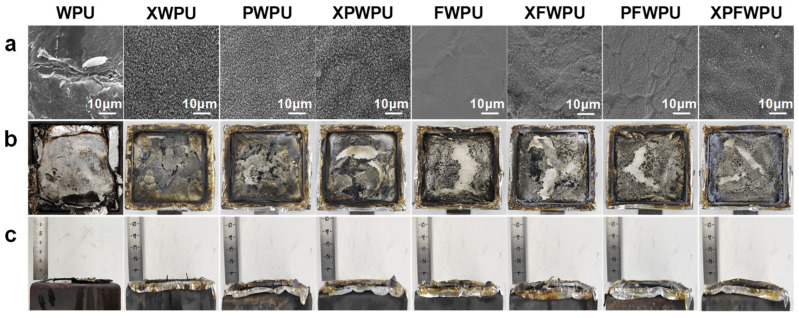
Surface micromorphology of char residue of the from cone calorimetry ((**a**), SEM-5000X); digital image of the char residue from the cone calorimeter front (**b**) and side (**c**).

In general, the morphology and structure of the char residue layer can reflect the flame retardant characteristics of WPU resins. During combustion, materials typically manifest in three distinct forms: fully burnt components (converted into heat and gasses), partially burnt elements (resulting in dripping and smoke), and the char residue layer itself. As shown in Figure 6, the morphologies of both neat WPU and its composites’ char residues after UL-94 vertical combustion tests were examined using SEM and optical photography. The char residues serve as effective insulators against heat and oxygen, thereby providing substantial protection to the WPU film. As shown in Figure 6a, it is evident that the char residue exhibited significant cracks and microholes from WPU to XPWPU. This contrasts sharply with the surface morphology observed in residues from FWPU to XPFWPU, which displayed a dense and uniform structure due to modification processes that enhance flame retardance within the WPU matrix materials. It is also noteworthy that the advanced performance of char residue formation aligns with that of WPU under nitrogen conditions. This indicates that both organoboron catalytic carbonization reactions and formed silicon dioxide–as an additive–react during combustion at rates comparable to those observed during thermogravimetric analysis at a programmed heating rate.

A high concentration region (bright area) for silicon elements is presented in Figure S3, which aligns with the distribution of white materials shown in Figure 6a. From Figure 6b,c and Figure S3, it can be concluded that the char residue primarily consists of carbon, SiO_2_, and boron oxides; newly formed SiO_2_ particles act as fillers during the formation process of char residue.

The char residue observed on FWPU films was consistent across samples—dense and devoid of white solids on their surfaces—supporting previous conclusions drawn from Figure 6a. As depicted in Figure 6b, the char residue on the surface of modified films appeared more intact compared to that seen in highly fragmented neat WPU residues. This indicates that polydimethylsiloxane segments significantly enhance the char-forming capacity of WPU due to migration processes involving Si-O bonds leading to silicon dioxide particle formation during combustion. Furthermore, enhancements were noted regarding film-forming capabilities within a WPU framework, alongside improved smoke suppression and flame retardant performance [25,26]. The incorporation of boron phenolic resin into WPU tendentially facilitates glassy carbon formation characterized by a dense structure throughout combustion processes; this results in a more cohesive and compact film structure upon burning. As demonstrated in Figure 6c, it has been confirmed that polydimethylsiloxanes combined with boron phenolic resin collaboratively promote carbonization within the WPU matrix. Uniform and dense char residues not only inhibit combustible release but also further isolate and protect internal WPU materials [27]. The resulting dense carbonaceous layer effectively restricts mass transfer as well as heat and oxygen flow.

It is evident that the carbon residue char layer enhances the protective effect and reduces the release of fuels into the gaseous phase. The Raman spectra of char residue were utilized to investigate the degree of graphitization in both neat waterborne polyurethane (WPU) and cured WPU during the combustion process. The results are presented in Figure S3. Two principal bands are observed at approximately 1600 cm^−1^ and 1350 cm^−1^, referred to as the G-band and D-band, respectively. The D band corresponds to a disordered graphite lattice structure, which includes edges, defects, surface layers, and a characteristic peak associated with Csp^3^ hybrid orbitals resulting from disordered arrangements of carbon atoms or lattice defects among nearest neighbors. In contrast, the G band pertains to an ideal graphitic lattice within the char residue layer, indicating an ordered lattice structure arising from vibrations of Csp^2^ in this layer. The relative intensity of the D-band reflects disorder within the crystal structure.

Generally speaking, the intensity ratio of the D-band to the G-band (I_D_/I_G_) is commonly employed to assess crystal disorder defect density and graphitization degree in residues. As is shown in Figure S3, a lower I_D_/I_G_ value signifies more graphitized structures, which contribute positively by enhancing heat insulation properties while reducing oxygen transfer through char residues.

The SEM-EDS was further employed to check the char residue after the cone calorimeter, the mechanism of the cured film and its structure in the flame retardant performance of WPU, and the distribution of C, O, N, Si, and B elements; the process of carbonization was also studied, as shown in Figure S4. Compared with the distribution of the elements on the surface of the cured film, the content of each element on the surface of the film in Figure S4 was gradually enhanced, and the color gradually deepened, and the distribution tended to be uniform. Compared with the distribution of Si elements in Figure S4(a1–b2), it can be seen that the Si elements were more abundant and uniform on the surface of char residue originated from the cooperatively cured film than that of the unilaterally cured film or neat WPU film, which also reflects that the ‘Y-type’ structured X-22 was more inclined to migrate to the surface than that of the straight-chain structured PDMS. The distribution of the surface elements of the film before and after combustion in Figure S4(a1–g2) was clearly observed, the Si and B elements were gathered on the surface after combustion, and the distribution characteristics of C, O, and N elements were consistent with each other. From the TG-DTG analysis results, it can be seen that the Si-O and B-O bond were formed in the second pyrolysis stage, and can promote the formation of WPU in a uniformly dense char residue layer. Simultaneously, it was confirmed that the WPU has been successfully modified by X-22, PDMS, and boron phenolic resin.

To investigate the impact of polydimethylsiloxanes and borophenolic resin on the flame retardancy of WPU with varying structures, the flame retardant and anti-dripping properties of the film were evaluated by LOI test and UL-94 vertical combustion test [28]. The UL-94 vertical burning test is an important method to evaluate the flammability and dripping hazards of materials. In this case, the various WPU films were tested, the LOI values and UL-94 grades of the WPU films were listed in Table 3, respectively. Obviously, the flame retardance and anti-dripping performance of the WPU cured with boron phenolic resin was effectively improved. The LOI value of pure WPU was only 21.9%, and the LOI value of FWPU was enhanced to 27.9%. The LOI values of the neat WPU, FWPU, XFWPU, PFWPU, and XPFWPU were 21.9%, 27.9%, 29.5%, 30.8%, and 32.6%, respectively. The UL-94 levels of the neat WPU film were unrated, the film cured with polydimethylsiloxanes were V-2 grades, the FWPU, XFWPU, and PFWPU films were all V-1 grades, and the XPFWPU film reached V-0 grade. It is generally believed that materials exhibiting LOI values above 26% would show self-extinguishing behavior in air and be considered to possess high flame retardancy. The LOI values of the FWPU, XFWPU, PFWPU, and XPFWPU fall into this category.

**Figure 7:**
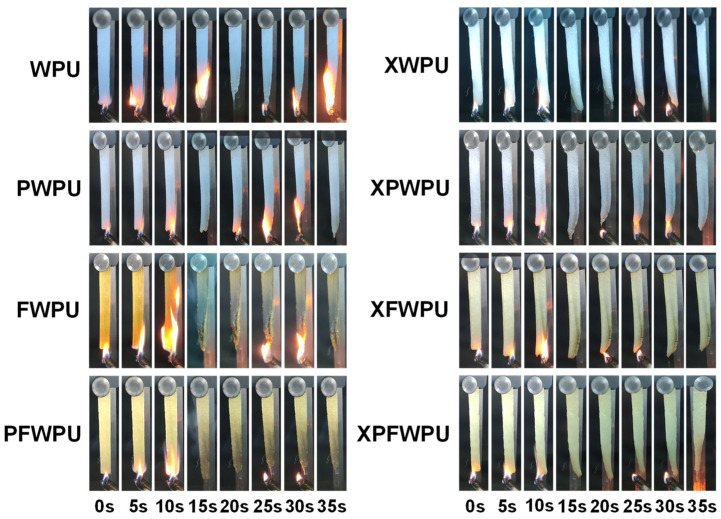
Vertical burning photo of the neat WPU film and the cured film, XWPU, PWPU, and XPWPU, FWPU, XFWPU, PFWPU, and XPFWPU.

The neat WPU, as a highly flammable polymer, was easily ignited and generated continuous melt drips during combustion (Figure 7). It can be seen from Figure 7 that in the drip test, the neat WPU film and the cured film (XWPU, PWPU, and XPWPU) are dripped during the combustion process. However, for the neat WPU film modified with polydimethylsiloxanes and boron phenolic resin, the anti-melt dripping effect of the film was significantly enhanced, and the FWPU, XFWPU, PFWPU, and XPFWPU films are non-dripping. It can be inferred that boron phenolic resin played an important role in improving the flame retardance, anti-dripping, and self-extinguishing performance. The WPU film cured with two type structures of polydimethylsiloxanes, were considered as good performance in anti-dripping and self-extinguishing, compared with that of the boron phenolic resin.

### 2.9. Proposed Flame Retardancy Mechanism

The mechanism is based on the main effects (B-O bond in Figure 1, WPU matrix char residue yield in Figure 3, and B element in Figure S4). As shown in Figure 3a, the char residue yield of the cured WPU can reach as high as 8.93%, which shows good flame retardancy with 32.6% LOI, non-dripping, and UL-94 V-0 grade than that of the neat WPU. In comparison, as shown in Figure 7, the neat WPU sample is almost burned out, and just leaves fragile and broken char residue layers. Meanwhile, there are many micro-holes and cracks on the char residue on the surface, leading to superior mass and heat transfer and inferior flame retardancy. As shown in Figure 7 with the introduction of the flame retardant, the morphologies of char residue layers for XFWPU, PFWPU, and XPFWPU samples have been significantly improved, which become dense and serve to protect the underlying WPU matrix, providing isolation from the fire during combustion. Supposedly, the flame retardant boron phenolic resin can promote the formation of a glassy state and continuous char residue from WPU matrix during the combustion progress.

The char residue formed was attributed to the synergistic carbonization of silicone–oxygen and boron phenolic moieties and the enhanced barrier effect of the WPU residual char layer with high graphitization and integrity quality. The boron phenolic moieties’ covalently grafted WPU component degrades at a lower temperature than that of the silicone–oxygen in the glassy char residue, induced by B-OH groups. On the one hand, the silicone–oxygen moieties can be transformed into SiO_2_ particles during the combustion process; on the other hand, the boron phenolic moieties can be employed to form a network structured WPU because of the reaction between the triple -OH groups and -NCO group. It is notable that the cross-linked network-structured WPU and benzene ring are collaboratively conducive to the promotion of its melt viscosity, melt strength, anti-dripping, and carbonization process. Both the network-structured WPU and organoboron collectively contribute to smoke suppression because of their capture effect regarding small diffusing particles which come from the smoke. It can be inferred that char residue layer plays an important role in delaying the ignition of the underlaid WPU matrix polymer materials by creating a complete physical blockade between the unburnt WPU matrix polymer materials and the flame. At the same time, the glassy char residue coated with SiO_2_ provides an active gas barrier effect by limiting oxygen access to the unburnt WPU matrix’s polymer materials and by averting the outflow of released gasses from the flame zone, including toxic fumes and flammable volatiles, which results in considerable reduction in TSP, SPR, AEHC, and eventually pHRR.

### 2.10. Analysis of Mechanical Properties of the WPU Film

**Figure 8:**
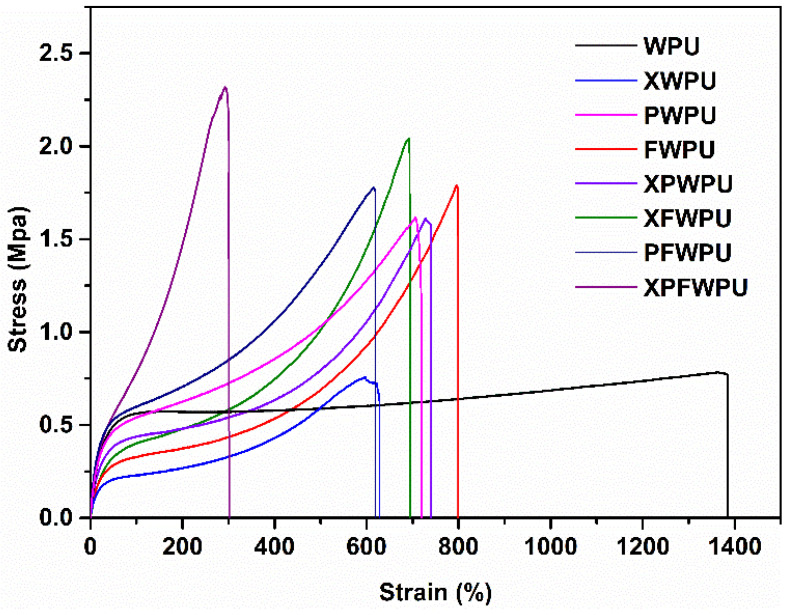
The stress–strain curves of the neat WPU and the cured WPU (PWPU, XWPU, XPWPU, FWPU, PFWPU, XFWPU, XPFWPU).

As is shown in Figure 8, the tensile strength and breaking elongation can be regulated in a large range. The tensile strength was enhanced by around 4 times and breaking elongation was reduced by about 11 times. The stereo-hindrance and winding function of the linear and ‘Y-type’ polydimethylsiloxanes chains and can be easily molded due to the network architecture, which can graft, twine, and hinder the WPU chain.

The hydroxyl linear chain structure of polydimethylsiloxanes can disperse the action of forces in the material, form an equivalent for the physical cross-linking point, and transmit the tensile stress between molecular chains. During the weight-bearing process, when the molecular chain is fractured, the WPU skeleton structure can still be maintained [29,30,31], thereby increasing the tensile strength of the modified WPU. The chain segment with Si-O has a certain degree of suppleness and flexibility, and as the chain lengthens, so does its tensile strength, and the Y-type structure of X-22 can lead to a short chain length, as involved in the construction of the WPU skeleton, so that the tensile strength is not significantly improved. Compared with the PDMS, the B-O chain segment involved in the construction of the WPU skeleton, the boron phenolic resin, has stronger mechanical properties. The WPU film cured with boron phenolic resin can disperse the stresses and mechanical stresses. However, the Si-O chain segment may lead to the microphase separation phenomenon and brittleness of the film [31]. Therefore, an appropriate introduction of polydimethylsiloxanes and the FB88 can be employed to regulate the tensile strength and the brittleness of the film.

### 2.11. Surface and Fracture Section Morphology of the WPU Film

**Figure 9:**
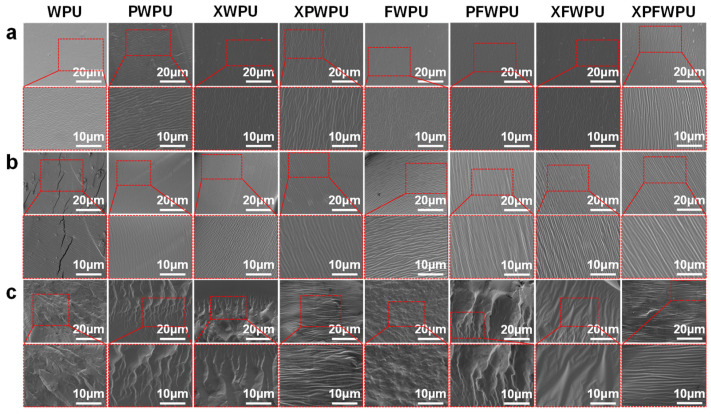
SEM images of the neat WPU and the cured WPU (PWPU, XWPU, XPWPU, FWPU, PFWPU, XFWPU, XPFWPU) film surface (**a**), the normal tensile section of film (**b**), the brittle section of the film (**c**).

The surface morphology of the char residues after the cone calorimetry test were carried out by SEM is shown in Figure 9, where it can be seen that the surface of the WPU film was rough and similar to a microscopic ravine. For the WPU film modified with polydimethylsiloxanes, the furrows on the surface of the film were obviously more regular and fine, while the surface ravines of the film were arranged in a mesh after boron phenolic resin modification. For the WPU film modified with polydimethylsiloxanes and boron phenolic resin, the fine lines on the surface of the film tend to be regularized, and cause phase separation of the soft and hard segments of the WPU [32,33]. As can be seen from Figure 9b, there were obvious cracks in the drawing section of the neat WPU, the cracks in the section were observed to have disappeared more frequently and extensively in the case of the neat WPU compared to other samples, and the microscopic morphology was smoother. As can be seen from Figure 9c, the brittle section of the WPU film had uneven cracks distributed irregularly, while the brittle section of the modified film was more regular and without obvious cracks. The results show that with the smoothness and regularity of the fractured section of the film surface, normal stretching and liquid nitrogen embrittlement fractures were improved significantly, and the permeability was significantly reduced. Because of the modification of WPU emulsion, the film-forming ability becomes stronger, and the cracks formed in film were significantly reduced. It was not easy to form a large area of layered cracks. At the same time, the lubricity of the emulsion was enhanced by modification, and the impact and fracture force of normal pulling and brittle breaking was weakened. And, it was not easy to form a large area of layered folds.

Based on Section 2, aspects which were once flaws of the WPU, such as its flammability, have been overcome. As a result, WPU materials have attracted growing interest, due not only to concerns about healthcare, heightened environmental awareness, and relevant laws and regulations, but also because of their robust adhesion and elasticity properties. WPU materials are widely used in various fields, including in coatings, adhesives, and surface treatment agents.

## 3. Materials and Methods

### 3.1. Materials

Polypropylene glycol 1000 (PPG-1000, industrial grade), isophorone diisocyanate (IPDI, analytical purity) and dimethylollapropionic acid (DMPA, analytical purity), 1,4-butanediol (analytical purity), and dibutyltin dilaurate (chemical purity) were purchased from Shanghai McLean Biochemical Technology Co., Ltd. (Shanghai, China). Acetone and triethylamine (analytical purity) were obtained from Chengdu Jinshan Chemical Reagent Co., Ltd. (Chengdu, China). Boron phenolic resin (FB88, Mw = 600, industrial grade) was provided by Bengbu Tianyu High Temperature Resin Material Co., Ltd. (Dongguan, China). PDMS (Mw = 4200, industrial grade) was provided by Alfa Aesar Chemical Co., Ltd. (Shanghai, China). X-22-176DX (abbreviated as X-22, Mw = 3600, industrial grade) was acquired from Xinyue Silicone International Trading (Shanghai, China) Co., Ltd. Acetone was administered with 4 Å type molecular sieves for further use. Other reagents were commercially available and implemented without any purification.

### 3.2. Preparation of the Emulsion

WPU emulsion was synthesized by self-emulsification. The emulsion was prepared using the acetone method. A total of 10.0 g (0.010 mol) PPG-1000 and 5.0 g (0.023 mol) IPDI, 0.2% of the prepolymer mass (in terms of IPDI and PPG-1000 total mass), were added into a four-necked round-bottom flask with a reflux condenser, a mechanical stirrer, a thermometer, and a nitrogen inlet. It was heated to 70 °C for 1 h and stirred at a rate of 300 rpm for 3 h. After the -NCO value reached the theoretical amount (di-n-butylamine backdropping method), DMPA 0.6358 g (0.0047 mol) and 1,4-butanediol 0.225 g (0.0025 mol) were, respectively, added into the flask and sufficiently mixed with the prepolymer to initiate the chain-growth extension reaction. The reaction proceeded for 2 h at 85 °C, and the reaction temperature was rapidly cooled to 55 °C.

**Figure 10:**
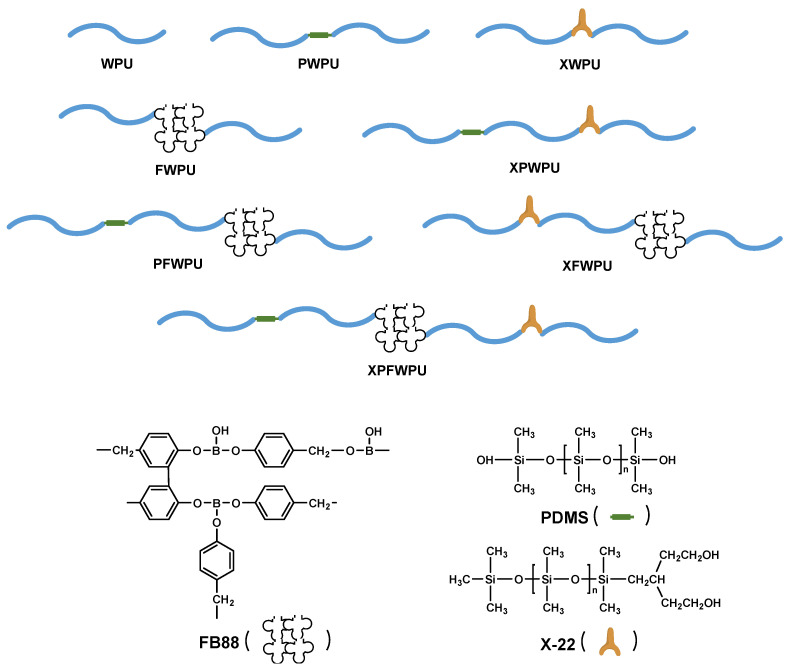
Schematic diagram of the novel flame retardant for the WPU.

In the same way, the flame retardant (2.0 wt.%, based on the IPDI and PPG1000 total mass) was added into the reaction system. Triethylamine 0.4756 g (0.0047 mol) with DMPA was used to neutralize the reaction system for 15 min, and the acetone was added to adjust the viscosity of the system, and the system was cooled to 25 °C, and 40 mL deionized water was added to the system to obtain emulsion, at high speeds for 30 min (1600 r/min). Reduced pressure distillation was carried out to remove the organic solvent acetone; that is, to obtain the purified and stabilized emulsion. The schematic illustration of this synthesis is shown in Figure 10 and is shown in Table S1, respectively.

### 3.3. Preparation of the WPU Materials

The emulsion was coated on the tinplate sheet surface, and naturally cast into a film on the horizontal surface at room temperature, and cured for 5 d. The maximum gel content values in the emulsion were evaluated to be approximately 35.0 wt.% by water evaporation. The samples were used to prepare the various WPU film materials. The emulsion was degassed and poured into a dumbbell-shaped PTFE mold to prepare the unique shape materials. Then, the mold was subsequently put in vacuum-drying oven at 50 °C for 5 d until the weight of the samples was obtained as a constant weight. Specifically, the emulsion was free of bubbles before vacuum drying. The film was removed from the mold and stored in a desiccator.

### 3.4. Measurements

#### 3.4.1. Fourier-Transform Infrared Spectroscopy (FTIR)

FTIR spectra were obtained on a Nicolet 6700 FTIR spectrometer (Thermo Fisher Scientific Inc., Waltham, MA, USA), using the KBr tablet method (KBr was previously oven-dried to avoid the interference of water). The measurements were carried out on thin disks prepared in a hydraulic press and spectra recorded. The scanning frequency was 64 times, and the wavenumber range was 500–4000 cm^−1^ and the resolution was 1 cm^−1^, with a signal-to-noise ratio of 30,000:1.

#### 3.4.2. The Emulsion Properties

One or two drops of emulsion were added to distilled water and diluted by a factor of one thousand. The particle size was determined to be 25 °C over a period of 120 s, utilizing a Malvern wet laser particle size meter (Malvern Panalytic, Malvern, UK) to evaluate the particle size and distribution of the emulsion. The WPU emulsions were subjected to a shear viscosity test using an NDJ-4 rotary viscometer(Shanghai Cany Precision Instrument Co., Ltd., Shanghai, China) with a No. 3 rotor at 25 °C, in accordance with GB/T2794-1995 (determination methods for viscosity of adhesives, Beijing, China). As per GB/T6753.3-1986 (methods of test for package stability of paints, Beijing, China, 1986), the emulsion was transferred to a centrifuge tube and centrifuged at 3500 r/min for 10 min. The absence of precipitation indicated that the emulsion could be stably stored for 6 months.

#### 3.4.3. Determination of Water Absorption and Water Resistance of the Films

Moreover, the water contact angle test was employed to detect the amphiphilicity property of the WPU modified with flame retardants. The water contact angle of the WPU films were evaluated by dripping deionized water onto the surface of the WPU films. The static drop method was employed to test the water contact angle. The droplets (one droplet about 4.0 μL) were then flushed into the curing unit. The average contact angle was obtained from five tests of each sample.

The water resistances of the cured films were evaluated by water absorption tests. The test was conducted with the same film quality, which was placed in water for 24 h and the residual water absorbed by filter paper, according to Equation (1), and the film’s water absorption rate was calculated: in the formula, *ω* was the water absorption rate used to calculate the film’s water absorption rate (%), and m_1_ and m_2_ were the mass of the film before and after immersing in the water.

(1) $$\omega \%=\frac{m_{2}-m_{1}}{m_{1}}\times 100\%$$

#### 3.4.4. Scanning Electron Microscope (SEM) Testing and Char Residue Analysis

Gold powder was applied to the coating surface to enhance conductivity. Liquid nitrogen was utilized to cool and embrittle samples. Scanning electron microscopy (SEM) was conducted to examine the surface morphology of the films and their char residues. The Czech TESCAN MIRA LMS scanning electron microscope–energy dispersion spectrometer (SEM-EDS, TESCAN Group a.s., Brno, The Czech Republic) was employed to observe the microscopic morphology and elemental distribution of the film residue at 15 kV. The elemental analysis of the char residues was characterized by SEM equipped with EDS-mapping.

#### 3.4.5. Thermogravimetric Analyzers

Thermogravimetric analysis (TG) of the cured films was conducted utilizing a NETZSCH (TG 209, NETZSCH-Gerätebau GmbH, Bavaria, Germany) thermogravimetric analyzer, employing the integral mass balance method to analyze mass changes with temperature during heating. The TG program was initiated by purging with nitrogen for 15 min to remove adsorbed impurities. A sample mass of 10.0 mg was utilized, and measurements were performed in duplicate. Following pulverization and extrusion into fragments, the samples were placed in an alumina crucible and heated from 30 °C to 600 °C at a heating rate of 10 °C/min under N_2_ flow. N_2_ (>99.99% by volume) served as the carrier gas. Char residue yield is calculated based on Equation (2).

(2) $$char\;residue\;\%=\frac{m_{4}}{m_{3}}\times 100\%$$

where m_3_ and m_4_ refer to initial and final mass at reference temperatures of 30 °C and 600 °C, respectively.

#### 3.4.6. Anti-Dripping Behavior of the WPU Film

All specimens were measured five times. All the samples were fabricated by the standard dimensions and requirement [34,35,36].

#### 3.4.7. Mechanical Properties Test

According to the tensile strength of the cured spline was evaluated on the XWW-20A universal testing machine (Chengde Donghai Testing Machine Manufacturing Co., Ltd., Chengde, China) [37]. The selected load was 500 N and the tensile rate was 3 mm/min. The specimen sizes were 25.0 × 5.0 × 5.0 mm^3^. The tensile properties of the sample were the average of the five measured values.

## 4. Conclusions

The surface properties of the WPU treated with flame retardant were enhanced. The water resistance of the WPU treated with polydimethylsiloxanes was enhanced compared to that of the boron phenol resin. The WPU treated with flame retardant has two pyrolysis stages; the difference of *T*_10%_ was not obvious. The *T*_50%_ increase in the cured WPU was improved from 3.1 °C to 48.0 °C. The thermal stability and char residue of the cured WPU were significantly enhanced.

The cured WPU had a significantly enhanced carbonization capacity, flame resistance, and anti-dripping performance. The ignition time could be extended to 24 s. Regarding the combustion strength and heat release, the CO/CO_2_ value was 0.0278, an increase of 46.32%; the total flue gas emission was cut down by 66.29%, and the smoke production rate was significantly reduced. It was shown that the WPU modified with polydimethylsiloxanes and boron phenolic resin was cooperatively endowed with good flame retardancy, anti-melt droplets, and flue gas inhibition. The cured WPU can reach 2.32 M Pa, which was about three times than that of the neat WPU.

## Figures and Tables

**Table 1:** The decomposition data of WPU the film.

Sample	*T*_10%_ (°C)	*T_5_*_0%_ (°C)	*T*_maxⅠ_ (°C)	*T*_maxⅡ_ (°C)	Char Residue Yield (wt.%)
WPU	275.3	321.7	302.4	357.1	0.15
PWPU	277.6	324.8	307.4	361.2	0.34
XWPU	292.5	345.3	312.3	367.3	0.68
FWPU	289.8	349.2	317.0	372.0	1.65
XPWPU	292.8	341.5	333.0	373.9	1.55
PFWPU	293.7	352.1	335.5	377.2	1.54
XFWPU	287.4	357.8	338.5	390.0	2.01
XPFWPU	278.9	369.7	348.7	391.3	8.93

**Table 2:** The cone calorimetry data of the WPU films.

Sample	TTI (s)	pHRR (kW·m^−2^)	THR (MJ·m^−2^)	ACOY (kg·kg^−1^)	ACO_2_Y (kg·kg^−1^)	CO/CO_2_	AEHC (MJ·kg^−1^)	TSP (m^2^)
WPU	5	1214.93	114.41	0.052	2.71	0.0190	45.53	5.37
XWPU	9	1353.55	101.01	0.086	3.75	0.0229	31.47	4.86
PWPU	11	1378.46	108.56	0.068	3.57	0.0192	29.64	4.05
FWPU	18	1295.61	95.67	0.087	3.66	0.0235	28.05	3.91
XPWPU	13	1292.22	97.43	0.090	3.93	0.0230	27.40	3.90
XFWPU	20	1114.30	93.60	0.092	3.92	0.0237	25.39	3.50
PFWPU	22	1039.04	85.51	0.100	3.95	0.0253	24.09	3.41
XPFWPU	25	875.24	82.36	0.138	4.97	0.0278	23.27	1.81

**Table 3:** LOI value and UL-94 rating of the neat and cured WPU film.

Sample		Vertical Burning Test			
		After-Flame Time			
	LOI (%)	$\bar{t_{1}}$ (s)	$\bar{t_{2}}$ (s)	UL-94 Rating	Dripping
WPU	21.9	76.0	78.2	unrated	yes
XWPU	23.2	31.3	30.7	V-2	yes
PWPU	23.7	28.6	27.5	V-2	yes
XPWPU	24.1	24.7	25.3	V-2	yes
FWPU	27.9	12.9	11.6	V-1	no
XFWPU	29.5	10.4	9.7	V-1	no
PFWPU	30.8	6.5	7.2	V-1	no
XPFWPU	32.6	4.2	4.8	V-0	no

Note: The flame of specimen burned to the clamp in the first flame application.

## Data Availability

The original contributions presented in the study are included in the article/Supplementary Materials; further inquiries can be directed to the corresponding authors.

## Supplementary Materials

The following supporting information can be downloaded at: https://www.mdpi.com/article/10.3390/molecules29235799/s1, Figure S1: Particle size and distribution status of emulsion; Figure S2: Film DSC curve; Figure S3: Raman spectra of the char residues of FWPU (a), XPWPU (b), XFWPU (c), PFWPU (d), XPFWPU (e), summary sheet of (a–e) (f); Figure S4: Surface topography and element distribution of a1-g1 and a2-g2, XWPU, PWPU, XPWPU, XFWPU, PFWPU, XFWPU tapered calorimetry film (SEM-5000X); Table S1: Composition of the different samples; Table S2: WPU and modified emulsion performance.

## Abbreviations

WPU	waterborne polyurethane
PWPU	WPU cured with polydimethylsiloxanes (PDMS)
XWPU	WPU cured with polydimethylsiloxanes(X-22)
FWPU	WPU cured with (Boron phenolic resin, FB88)
XPWPU	WPU cured with polydimethylsiloxanes (PDMS and X-22)
PFWPU	WPU cured with polydimethylsiloxanes (PDMS) and (Boron phenolic resin, FB88)
XFWPU	WPU cured with polydimethylsiloxanes(X-22) and (Boron phenolic resin, FB88)
XPFWPU	WPU cured with polydimethylsiloxanes (PDMS and X-22) and (Boron phenolic resin, FB88)
