# Supplementary material for: Waterborne Polyurethane Treated with Flame Retardant Based on Polydimethylsiloxanes and Boron Phenolic Resin for Improving the Char Residue and Anti-Dripping Performance
Source: Molecules. 2024 Dec 8;29(23):5799. doi: 10.3390/molecules29235799 (PMC11643660; doi:10.3390/molecules29235799)
Supplement: Supplementary file 1 [file molecules-29-05799-s001.zip › molecules-3295811-supplementary.pdf]

Supplementary Materials

Waterborne Polyurethane Treated with Flame Retardant Based on Polydimethylsiloxanes and Boron Phenolic Resin for Improving the Char Residue and Anti-Dripping Performance

Yadian Xie [1,*], Chao Liu [1], Yujie Wang [1], Dongmei Bao [1], Wei Yan [2], and Guoyong Zhou [1,*]

[1]*School of Chemical Engineering, Guizhou Minzu University, Guiyang 550025, PR China*

[2]*School of Materials Science and Engineering, Guiyang University, Guiyang 550005, PR China*

Table S1 Composition of the different samples

Sample	PPG1000 (g)	IPDI (g)	DMPA (g)	BDO (g)	PDMS (g)	X-22 (g)	FB88 (g)	TEA (g)	H_2O (ml)
WPU	10	5	0.6358	0.225				0.4756	35
PWPU	10	5	0.6358	0.225	0.3			0.4756	35
XWPU	10	5	0.6358	0.225		0.3		0.4756	35
XPWPU	10	5	0.6358	0.225	0.3	0.3		0.4756	35
FWPU	10	5	0.6358	0.225			0.3	0.4756	35
PFWPU	10	5	0.6358	0.225	0.3		0.3	0.4756	35
XFWPU	10	5	0.6358	0.225		0.3	0.3	0.4756	35
XPFWPU	10	5	0.6358	0.225	0.3	0.3	0.3	0.4756	35

Table S2 WPU and modified emulsion performance

Sample	Performance				
	Exterior	Particle size(nm)	PDI	Viscosity (mp·s)	Stable
WPU	Translucent, Blu-ray	66.33	0.119	21.5	*
FWPU	Translucent, Blu-ray	73.32	0.097	23.7	*
XWPU	Translucent, Blu-ray	77.16	0.108	24.6	*
PWPU	Translucent, Blu-ray	87.74	0.046	25.8	*
XFWPU	Yellowish translucent, Blu-ray	106.73	0.175	27.3	*
PFWPU	Yellowish translucent, Blu-ray	126.70	0.035	29.5	*
XPWPU	Yellowish translucent, Blu-ray	110.60	0.019	27.8	*
XPFWPU	Yellowish translucent, Blu-ray	147.52	0.198	31.9	*

(Note: * indicates stability, storage time > 6 months)

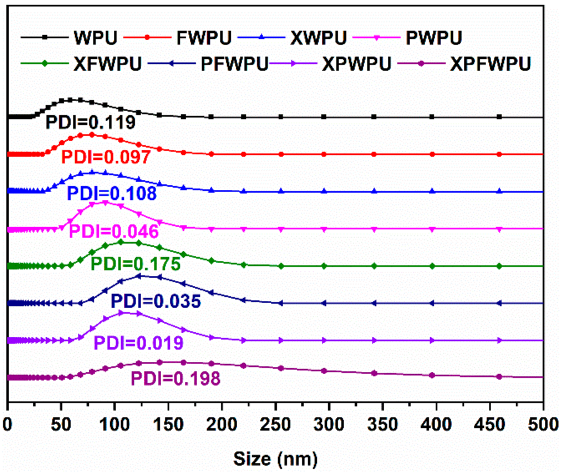

Figure S1 Particle size and distribution status of emulsion

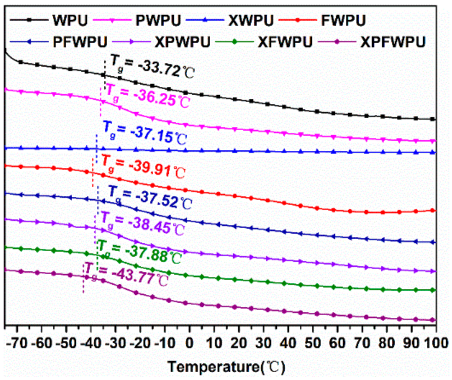

Figure S2 Film DSC curve

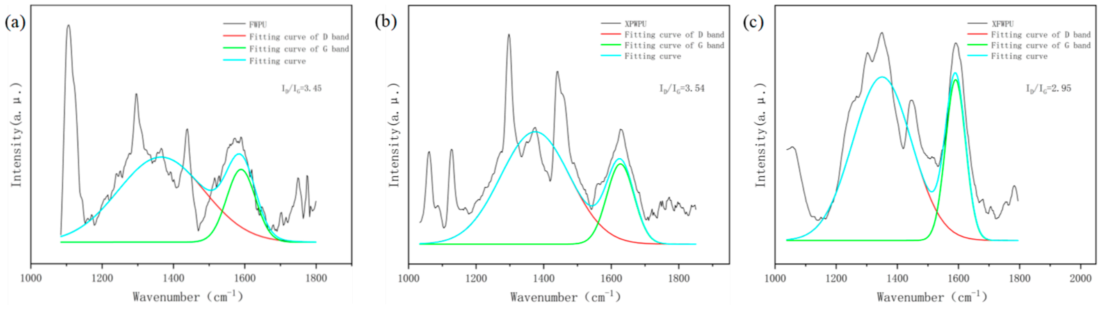

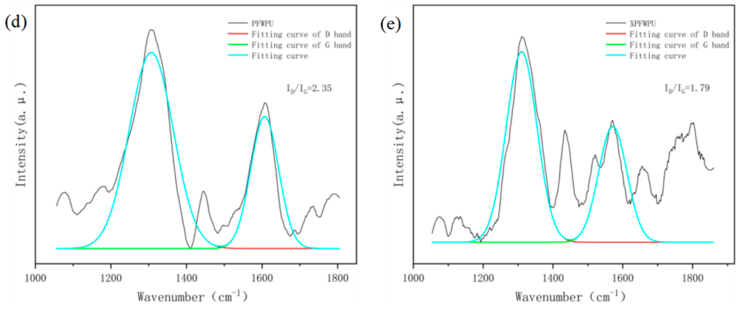

(f)

samples	ID/IG
FWPU	3.45
XPWPU	3.54
XFWPU	2.95
PFWPU	2.35
XPFWPU	1.79

Figure S3 Raman spectra of the char residues of FWPU (a), XPWPU (b), XFWPU(c), PFWPU (d), XPFWPU(e), summary sheet of a-e (f).

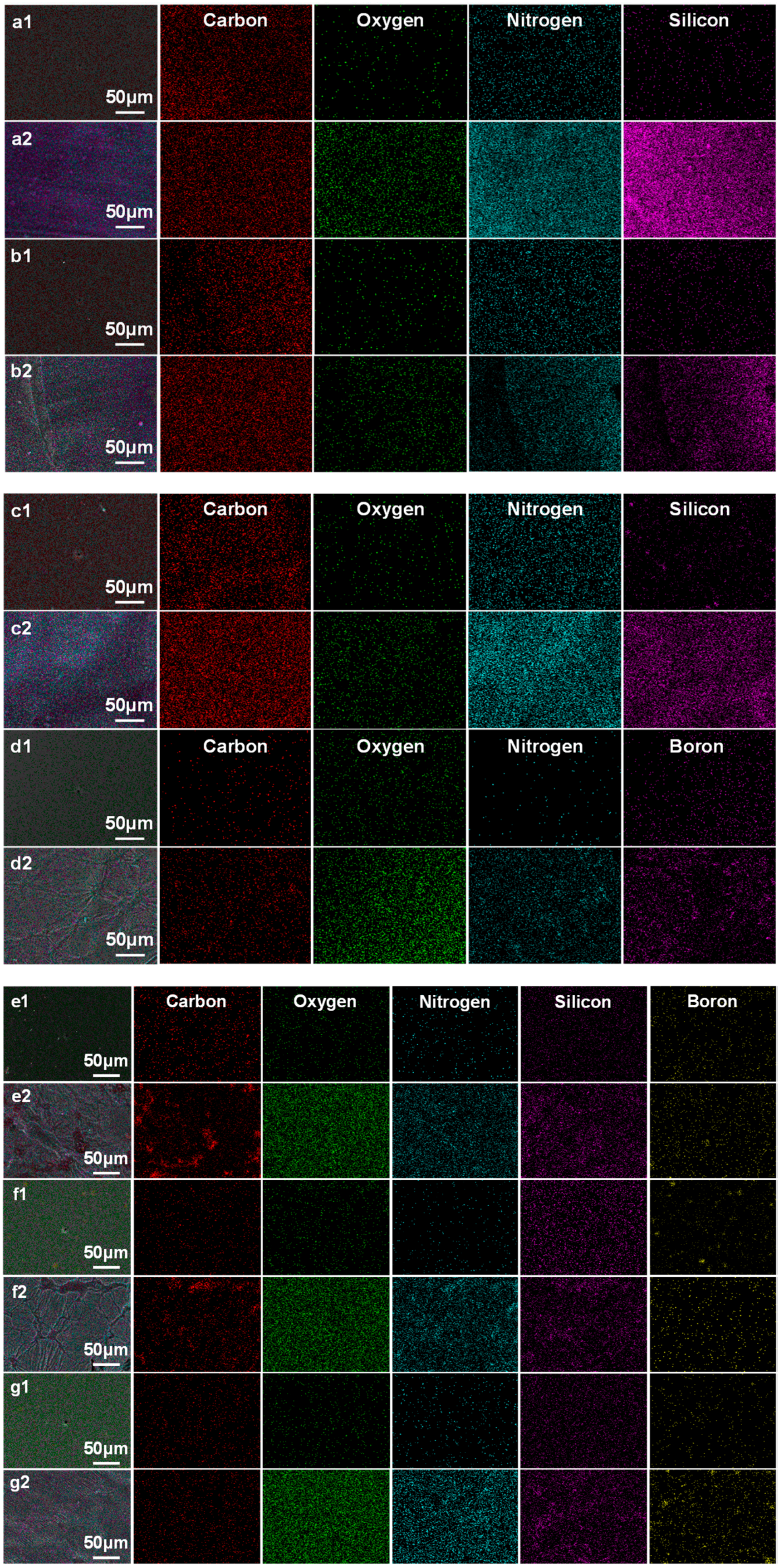

Figure S4 Surface topography and element distribution of a1-g1 and a2-g2, XWPU, PWPU, XPWPU, XFWPU, PFWPU, XFWPU tapered calorimetry film (SEM-5000X)
